# Spectrotemporal sound preferences of neighboring inferior colliculus neurons: implications for local circuitry and processing

**DOI:** 10.3389/fncir.2012.00062

**Published:** 2012-09-27

**Authors:** Chen Chen, Francisco C. Rodriguez, Heather L. Read, Monty A. Escabí

**Affiliations:** ^1^Department of Electrical and Computer Engineering, University of ConnecticutStorrs, CT, USA; ^2^Department of Biomedical Engineering, University of ConnecticutStorrs, CT, USA; ^3^Vicerrectoría Estudios de Postgrado, Universidad Don BoscoSan Salvador, El Salvador; ^4^Department of Psychology, University of ConnecticutStorrs, CT, USA

**Keywords:** spectrotemporal receptive field, laminar organization, receptive field transformation, correlated activity

## Abstract

How do local circuits in the inferior colliculus (IC) process and transform spectral and temporal sound information? Using a four-tetrode array we examined the functional properties of the IC and metrics of its micro circuitry by recording neural activity from neighboring single neurons in the cat. Spectral and temporal response preferences were compared for neurons found on the same and adjacent tetrodes (ATs), as well as across distant recording sites. We found that neighboring neurons had similar preferences while neurons recorded across distant sites were less similar. Best frequency (BF) was the most correlated parameter between neighboring neurons and BF differences exhibited unique clustering at ~0.3 octave intervals, indicative of the frequency band lamina. Other spectral and temporal parameters of the receptive fields were more similar for neighboring neurons than for those at distant sites and the receptive field similarity was larger for neurons with small differences in BF. Furthermore, correlated firing was stronger for neighboring neuron pairs and increased with proximity and decreasing BF difference. Thus, although response selectivities are quite diverse in the IC, spectral, and temporal preference within a local microcircuit are functionally quite similar. This suggests a scheme where local circuits are organized into zones that are specialized for processing distinct spectrotemporal cues.

## Introduction

Three organization principles for brainstem input to the central nucleus of inferior colliculus (ICC) are the basis of the “synaptic domain hypothesis” that predicts a division of ICC into synaptic and functional domains with unique sound processing capability (Oliver, 2000; Loftus et al., 2010). First, tonotopically organized inputs from the brainstem project onto banded anatomical lamina with spatially restricted dendritic arbors and common best frequency (BF), that in the cat can extend across a ~3.5 × 3.5 mm^2^ area (Morest and Oliver, 1984; Oliver and Morest, 1984; Serviere et al., 1984; Malmierca et al., 1993; Brown et al., 1997; Schreiner and Langner, 1997). Second, there is considerable convergence of different brainstem nuclei as well as different neuronal cell types onto a given frequency lamina that could allow for genesis of novel sound response properties (Adams, 1979; Oliver, 2000; Malmierca et al., 2005; Loftus et al., 2010). Third, brainstem projections are not uniformly distributed throughout all frequency laminae or even within a single frequency lamina (Oliver, 2000; Malmierca et al., 2005; Cant and Benson, 2008). These three anatomic patterns of pathway input to the ICC suggest a division of ICC into domains that process distinct sound features.

Though there is considerable evidence for anatomic domains, there is some debate regarding the structure of response property domains within ICC and how to demonstrate them. Frequency selectivity for single neurons shifts in spatial register with anatomically defined frequency laminae within the ICC (Schreiner and Langner, 1997; Malmierca et al., 2008). These BF response domains that we will refer to as, “frequency-band laminae,” can be resolved with micrometer precision along the dorso-ventral axis of ICC using single and multi-unit recording methodologies (Schreiner and Langner, 1997; Malmierca et al., 2008). Though it is clear that best frequencies are highly organized within and across laminae, how other response properties are locally organized within this laminar structure is less clear. Response property gradients for temporal modulations and spectral resolution have been shown to exist along the laminar dimension of the ICC (Schreiner and Langner, 1988; Ehret et al., 2003). Thus, it might be expected that neural response properties other than best frequencies should cluster within a local neighborhood so that neighboring neurons encode similar acoustic features. However, a recent study addressed this question and found that the tone-response properties of neighboring neurons were quite diverse (Seshagiri and Delgutte, 2007).

We examine how spectrotemporal response properties are organized and the degree of precisely correlated firing within a local ICC neighborhood. We used tetrode arrays to record and isolate neighboring single neurons and compared the spectrotemporal receptive preferences of each neuron pair. We demonstrate that neurons are highly organized within a local neighborhood where the receptive field similarities depend on both proximity and BF match. Furthermore, although correlated firing between neighboring neurons was generally low, it was more prevalent than expected by chance and the strength of correlated firing between neighboring neurons increased with local proximity. Thus, ICC is organized into distinct zones or neighborhoods with common spectrotemporal preferences that can effectively signal similar messages to the thalamus.

## Materials and methods

### Experiment procedure and acoustic stimuli

Animals were handled according to approved procedures by the University of Connecticut Animal Care and Use Committee and in accordance with National Institutes of Health and the American Veterinary Medical Association guidelines. Adult cats (*N* = 7) were initially anesthetized with a mixture of Ketamine (10 mg/Kg) and Acepromazine (0.28 mg/Kg I.M.) and were subsequently maintained in a surgical state with either sodium pentobarbital (30 mg/Kg, *N* = 2) or isoflurane gas mixture (3–4%, *N* = 5). The pinnae were retracted and the animal was placed in a stereotaxic assembly with hollow earbars. The Inferior Colliculus (IC) was exposed by removing the overlying cortical tissue and the bony tentorium. Following surgery, the animal was maintained in an areflexive state of by continuous infusion of Ketamine (2 mg/kg·h) and Diazepam (3 mg/kg·h), in a lactated ringers solution (4 mg/kg·h). Physiologic data (heart rate, temperature, breathing rate, and reflexes) was monitored to control the infusion rate. All neural recordings were performed over a period of 24–72 h.

Sounds were delivered in a sound-shielded chamber (IAC, Bronx, NY) via hollow ear-bars (Kopf Instruments, Tujunga, CA). The system was calibrated (flat spectrum between 200 Hz and 40 kHz, ±3 dB) with a Finite Impulse Response (FIR) inverse Filter (Implemented on a TDT® RX6 Multifunction Processor, Alchua, Fl). Sounds were delivered with a RME DIGI 9652 (Haimhausen, Germany) through dynamic speaker drivers (Beyer DT770). Dynamic Moving Ripple (DMR) sound was delivered dichotically in five experiments and monaurally to the contralateral ear in two experiments (Escabí and Schreiner, 2002). The DMR is a time-varying broadband sound (1–48 kHz; 96 kHz sampling rate; 100 spectral components/octave) that contains spectral (0–4 cycles/octave) and temporal (0–500 Hz) modulations that have been shown to efficiently activate ICC neurons and are prominent features in natural sounds (Rodriguez et al., 2010a). A 10-min sequence of the DMR was presented twice (Trial A and Trial B, 20 min in total) at fixed intensity (80 dB SPL, 65 dB spectrum level per 1/3 octave).

### Electrophysiology

**Figure 1:**
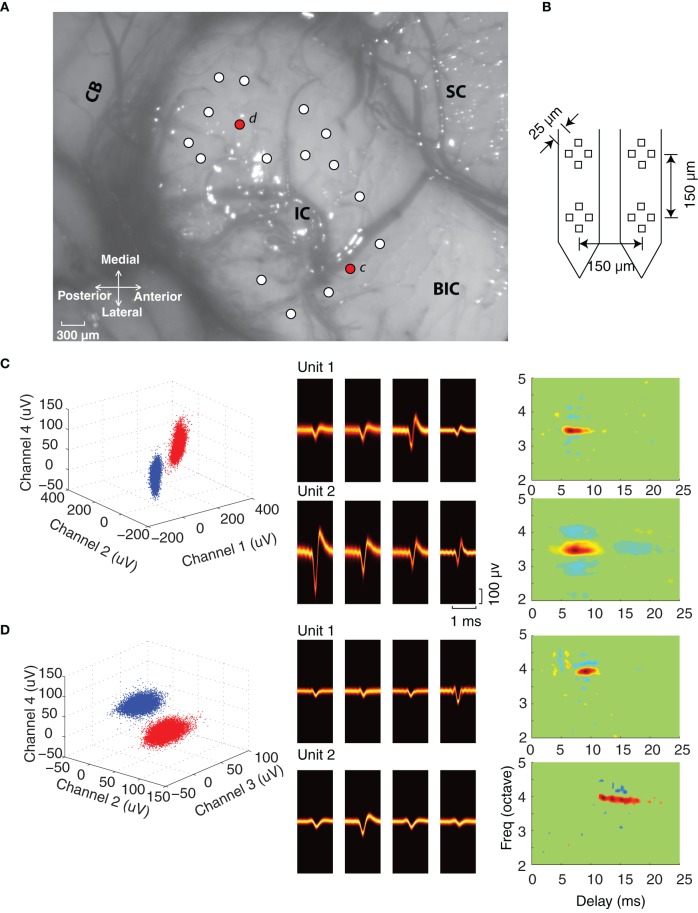
**Experiment configuration and single unit isolation. (A)** The dorsal–lateral surface of the exposed IC in one experiment along with the surrounding neural structures (SC, superior colliculus; BIC, brachium of the inferior colliculus; CB, cerebellum). Electrode penetrations are marked by circles (white and red) and were orientated an angle of 30° relative to the sagittal plane (roughly orthogonal to the frequency-band lamina). **(B)** Four-tetrode array used for the neural recordings. **(C,D)** Single unit isolation in two tetrode recordings. Each site has two well-isolated units as noted by the peak waveform amplitudes clusters at selected tetrode channels (blue and red clusters, *left*). The spike waveform density plots (*middle*) on each of the tetrode channels are shown for each of the isolated units. The waveform density represents the probability that the action potentials recorded on a given channel follow a particular waveform trajectory (color represents probability; orange is high, black is low), thus highlighting the most likely waveform pattern for each channel. Spectrotemporal receptive fields (STRF) are shown for each of the isolated units on the far *right*.

We used tetrode arrays (NeuroNexus Technologies, Ann Arbor, MI) to record neural activity from neighboring neurons in the ICC. The dorsal aspect of the exposed IC is shown from one experiment along with the penetration locations (white and red dots; Figure 1A). At each penetration location, a four-tetrode array was inserted (Figure 1B) and sound evoked neural activity was recorded. The tetrode array consists of four tetrodes (4 × 4 electrodes; two shanks with two tetrodes on each shank; impedance 1.5–3.5 MΩ at 1 kHz) with an inter-tetrode separation of 150 μm. This configuration is ideal for recording from neighboring neurons within and across adjacent frequency band laminae, which have a reported separation of ~150 μm in the cat (Rockel and Jones, 1973; Oliver and Morest, 1984).

The probes were first positioned on the surface of the IC with the assistance of a stereotaxic frame (Kopf Instruments, Tujunga, CA) at an angle of ~30° relative to the sagittal plane (~ orthogonal to the laminar orientation) (Merzenich and Reid, 1974; Semple and Aitkin, 1979; Schreiner and Langner, 1997). Electrodes were inserted into the IC with a LSS 6000 Inchiworm (Burleigh EXFO; Vanier, Quebec). Efforts were made to sample different regions of ICC by moving the electrode along the medio-lateral and rostral-caudal axis. Figure 1A shows a picture of the IC exposure from one of the experiments with the recording positions (white circles). At each penetration location we advanced the probe depth and recorded only from locations that followed a clear tonotopic gradient (Merzenich and Reid, 1974; Semple and Aitkin, 1979) and which exhibited well-isolated single units (sorted offline, see below). The probes were advanced until the end of the probe was reached (3 mm total length).

Neural responses were digitized and recorded with a RX5 Pentusa Base station (TDT®, Alchua, Fl) followed by offline analysis in MATLAB (Mathworks Inc., Natick, MA). The continuous neural traces were digitally band-pass filtered (300–5000 Hz) and the cross-channel covariance was computed across tetrode channels. Vectors consisting of the instantaneous channel voltages across the tetrode array that exceeded a hyperellipsoidal threshold of *f* = 5 (Rebrik et al., 1999) were used to detect candidate action potentials. This method takes into account across-channel correlations between the voltage waveforms of each channel and requires that the normalized voltage variance exceeds 25 units: *V*^*T*^*C*^−1^*V* > *f*^2^, where *V* is the vector of voltages, *C* is the covariance matrix, and *f* is the normalized threshold level. Spike waveforms were aligned and sorted using peak voltage values and first principle components with an automated clustering software (KlustaKwik software) (Harris et al., 2000). Sorted units were classified as single units only if the waveform signal-to-noise ratio exceeded 3 (9.5 dB, SNR defined as the peak waveform amplitude normalized by the waveform standard deviation), the inter-spike intervals exceeded 1.2 ms for >99.5% of the spikes, and the distribution of peak waveform amplitudes was unimodal (Hartigan Dip test, *p* < 0.05). These sorting criteria yielded 410 well-isolated single units from 280 tetrode recordings (an average of 1.46 well-isolated units per tetrode). The normalized separation between sorted units was ascertained by computing the Mahalanobis distance between the waveforms of two clustered units:

$$D_{1-2}=\sqrt{{\left({\bar{V}}_{1}-{\bar{V}}_{2}\right)}^{T}C_{12}^{-1}\left({\bar{V}}_{1}-{\bar{V}}_{2}\right)},$$

where ${\bar{V}}_{1}$ and ${\bar{V}}_{2}$ are the average waveforms for cluster 1 and 2 and *C*_12_ = (*C*_1_ + *C*_2_)/2 is the average covariance matrix of the spike waveform clusters. This quantity is unitless and represents the average separation between two clusters after normalizing by the average cluster spread. Distances between isolated clusters ranged from 2.3 to 22.1 (mean = 7.3) with most pairs above 3 (97%).

The interpretation of the tetrode recordings as they relate to the micro-circuitry of the ICC is critically dependent on the spatial separation between the recorded units. Thus it is desirable to define bounds for the distance between neighboring neurons in order to allow us to make inferences about functional organization. The recording radius of the tetrode array was estimated using the procedure outlined by Mechler and colleagues (Mechler et al., 2011). Briefly, the recording radius is estimated as

$$R=-\Delta s\cdot C_{T}\cdot \log\left(n\right)/\log\left(A\right)$$

where Δs = 28.5 μm is the average separation between the electrode contacts, *C*_*T*_ = 0.57 is a correction form factor that takes into account the geometry of the tetrode array (square geometry for the electrode contacts in our case), *A* is the contact-pair potential attenuation ratio (a number <1) which is defined as the average ratio of measured voltages across all contact pairs (*A* = 〈*V*_*i*_/*V*_*j*_〉 = 0.63) arranged so that *V*_*i*_/*V*_*j*_ < 1, and *n* = *V*_max_/*V*_min_ = 16.3 is an approximation of the signal-to-noise ratio where *V*_max_ and *V*_min_ is the largest and the smallest observed action potential amplitudes, respectively. Using this approach we estimated a recording radius of 97 μm for our recordings.

### Spectro-temporal analysis

Spectrotemporal receptive fields (STRFs) for the contralateral ear of identified ICC single neurons were obtained using spike-triggered averaging. Spectro-temporal parameters were obtained for each STRF according to procedure described previously (Rodriguez et al., 2010b). Briefly, we first determined the STRF power density by computing the STRF power at each time-frequency location, *p*(*t*, *x*) where *t* is time and *x* = log_2_(*f*/1000) is octave frequency (relative to 1000 Hz). The spectral and temporal power marginals were obtained by collapsing *p*(*t*, *x*) along the temporal and spectral dimensions and normalizing for unit area, respectively. Latency and BF were defined as the peak of the temporal and spectral power marginals; integration time and bandwidth were defined as twice the SD of the temporal and spectral power marginals, respectively.

The spectral and temporal modulation parameters of each unit were obtained directly from the ripple transfer function (RTF) by performing a two-dimensional Fourier transform of the STRF and subsequently computing the transfer function magnitude. The characteristic temporal and spectral modulation frequencies (cTMF and cSMF, respectively) were derived by computing the centroids from the modulation power marginal of the RTF (Rodriguez et al., 2010b).

### Sample selection and statistical analysis

Following the single unit isolation and the receptive field calculations, neural data was checked for stability. Due to the sound delivery paradigm, which consists of two 10-min repeats of the DMR, it is possible that adaptation or changes in anesthetic state could bias the results. To assure that neural response sensitivities were stable across consecutive trials we required that the STRF similarity index between the two DMR trials exceeded 0.5 (Escabí and Schreiner, 2002). This resulted in a reduction of the sample size from 410 to 344 single units, but assured that the receptive fields were stable across trials (mean SI = 0.71 prior to pruning; mean SI = 0.83 after pruning), thus minimizing any potential biases that could occur from the recording stability. For verification, we performed all of the analysis before and after removal of unstable neurons and the resulting trends and findings of the study were the same.

The neural data was next partitioned into three experimental and two control groups in order to make comparisons between neighboring and distant neuron pairs. The experimental and control groups were selected on the basis of the recording proximity between neighboring neurons and their BF difference. The first experimental group consisted of all neuron pairs that were isolated on the same tetrode (ST). A second group was defined for pairs of neurons that were isolated concurrently on adjacent tetrodes (ATs) but within the same recording site. Based on the estimated recording radius of 100 μm and an inter-tetrode separation of 170 μm, ST pairs are limited to ~200 μm separation while AT pairs are at most ~370 μm apart. Although, there is no guarantee that individual ST pairs are actually closer than individual AT pairs, on average ST pairs are closer to one another since the effective volume sampled by a single tetrode is substantially smaller than the effective volume sampled by ATs. Due to the fact that the average distance between ATs is 170 μm and the recording radius of each tetrode is ~100 μm, it is possible for two ATs to pick up the same unit at the same time. This was indeed the case for some AT pairs (14%) as evidenced by a strong single peak at the center bin of their spike train correlogram (i.e., at zero delay). For such sites, we kept the isolated unit with highest signal-to-noise ratio and discarded the accompanying unit from the AT. Finally, a third experimental group was defined to account for the fact that spectral and temporal response parameters such as bandwidth and latency are strongly correlated with the BF of neurons (Rodriguez et al., 2010b). Pairs from this third experimental group exist on the ST, but are chosen to be matched in BF (within 0.1 octave; same tetrode best frequency matched, STBF). Overall, the experimental groups consisted of 333 AT pairs, 93 ST pairs, and 54 STBF pairs.

Two control groups were defined as references for our statistical comparisons. As a null hypothesis, we require that spectrotemporal properties between neighboring neurons are uncorrelated and do not depend on proximity. The first control group was chosen by selecting neuron pairs from distant recording sites (DS group). Pairs from this control group were chosen at random from different recording sites and thus we expect that receptive field properties will be uncorrelated between selected pairs of neurons. A second control group was defined to account for the fact that certain spectral and temporal parameters are correlated with BF. For instance, latencies and bandwidth are both correlated with BF (Rodriguez et al., 2010b). Since, our intent is to identify response correlations that strictly depend on the proximity, and neighboring neurons are expected to have similar BFs, such dependencies need to be accounted for. The distant site BF matched (DSBF) control group was defined by selecting DS pairs that were closely matched in frequency (within 0.1 octave). The DSBF control group is thus necessary to account for any residual correlation that arises because two neighboring neurons share a similar BF, irrespective of proximity. Overall, the control groups consisted of 22,178 distant recording site pairs (DS) and 1963 DSBF pairs.

Statistical analysis was carried out on each of the receptive field parameters by comparing the control and experimental groups. For each receptive field parameter (e.g., BW, latency, etc.), the correlation coefficient was computed as a measure of similarity between the receptive field parameter of the selected neuron pairs from each group. Significant differences between temporal and spectral response properties between groups were identified by performing a Fisher z-transform test on the correlation coefficient. Four comparisons were made to characterize the effects of receptive field similarity and the dependence with proximity and BF match. First, DS vs. AT and AT vs. ST groups were compared to determine whether proximity has an effect on receptive field similarity. Next, we determined whether BF match has an effect on the receptive field similarity by comparing the ST vs. STBF groups. Finally, we controlled for the fact that some of the correlation observed as a function of proximity might be due to BF dependence and so we compared the STBF vs. DSBF groups. All tests were performed at a chance level of 0.05 with Bonferroni correction to account for the number of comparisons.

### Spike train crosscovariance

The shuffled crosscovariance (SCC) between the spike trains of single units was computed to evaluate the level of stimulus-driven response correlation (neuron 1 vs. neuron 2). The SCC between the spike trains of two neurons is defined as

$$\phi_{12}\left(\tau \right)=\frac{\phi_{1\text{A},2\text{B}}\left(\tau \right)+\phi_{1\text{B},2\text{A}}\left(\tau \right)}{2}$$

where 1 and 2 designates the neuron, A and B designates the stimulus trial. Here ϕ_XY_ (τ) = 〈 (*r*_X_(*t*) − λ_X_) · (*r*_Y_(*t* + τ) − λ_Y_)〉 is the spike train crosscovariance (i.e., crosscorrelation with means removed), *r*_X_(*t*) and *r*_Y_(*t*) are the response spike trains (units of spikes/s) sampled at 4 kHz sampling rate (250 μs bin width), and λ_X_ and λ_Y_ represent the average spike rates over the entire sound duration. The trial shuffling is performed to isolate stimulus driven correlations. The SCC was normalized as

$$C_{12}\left(\tau \right)=\frac{\phi_{12}\left(\tau \right)}{\sqrt{\phi_{1\text{A},1\text{B}}\left(0\right)\cdot \phi_{2\text{A},2\text{B}}\left(0\right)}}$$

so that −1 ≤ *C*_12_ (τ) ≤ 1. The spike train correlation index (CI) is defined as *C*_12_ = max[*C*_12_ (τ)]

Significance testing was performed by considering a random spike train with matched firing rate and interspike intervals as a null hypothesis. To do this, random spike trains were generated by shuffling the interspike intervals from the original spike trains from neuron 1 and 2. This procedure was repeated iteratively and the crosscovariance was computed for each iteration. The crosscovariance samples were used to estimate the mean and SE for the shuffled (null hypothesis) case. A *t*-test was then performed between the measured crosscovariance and the null hypothesis by requiring that the CI exceed chance level of *p* < 0.001 above (positive correlation) or below (negative correlation) the mean.

### Receptive field crosscovariance

A metric of receptive field similarity was defined to characterize the diversity of spectrotemporal features across the neural population. The receptive field crosscovariance (RFCC) function is first obtained by crosscorrelating the STRFs between two units (1 and 2) according to

$$\Phi_{1,2}\left(\tau ,\chi \right)=\iint {\text{STRF}}_{1}\left(t,x\right)\cdot {\text{STRF}}_{2}\left(t+\tau ,x+\chi \right)dtdx.$$

where τ and χ are temporal and spectral delays, respectively. The normalized RFCC is obtained as (Chen et al., 2012):

$${\bar{C}}_{12}\left(\tau \right)=\frac{\Phi_{12}\left(\tau ,0\right)}{\sigma_{1}\cdot \sigma_{2}}$$

where σ^2^_1_ and σ^2^_2_ correspond to the STRF power (i.e., σ^2^_*k*_ = ∫∫STRF_*k*_(*t*, *x*)^2^ *dtdx*). The RFCC corresponds to the normalized covariance between the predicted responses of two neurons under the assumption that each of the neurons behaves linearly. Thus the RFCC serves as a linear model prediction for the SCC. Values near one indicate that the receptive fields of the two units and, hence, the predicted spike rate patterns, are identical while values near zero indicate that the receptive fields and predicted spike trains are uncorrelated. Finally, it can be shown that the magnitude of the RFCC sets an upper bound on the magnitude of the spike train crosscovariance:

$$\left|{\bar{C}}_{12}\left(\tau \right)\right|\geq \left|C_{12}\left(\tau \right)\right|$$

where equality holds for the case of linear processing This upper bound strictly holds under the assumption that neural variability and non-linearities are independent between the two neurons tested. Although, this may not strictly hold, we've demonstrated that the upper bound holds for most neuron pairs tested in the ICC (Chen et al., 2012).

## Results

We used tetrode arrays to compare the spectrotemporal response properties of neighboring neurons in the ICC (Figure 1B). We asked whether neighboring ICC neurons have similar spectrotemporal preferences and how receptive field properties vary with BF and proximity within a local neighborhood. A concerted effort was made to thoroughly sample the rostral-caudal and medio-lateral dimensions of the ICC (as evident from Figure 1A) to assure that neurons with a diverse repertoire of spectral and temporal properties were included in the sample.

The effective size of the recording neighborhood for the tetrode array was estimated with the triangulation method of Mechler et al. (2011) (see Materials and Methods). We assume that the measured action potential voltage decays as a power-law with distance from the source and that the rate of decay depends on the tetrode geometry, impedance and the electrical characteristics of the surrounding tissues. For our configuration, the tetrode electrodes are arranged in a square with an inter-electrode separation of 25 μm on the sides, so that the average separation between electrodes is 28.5 μm (after considering diagonals). Based on the observed maximum SNR (16.3) and the average inter-electrode attenuation ratio (0.63) we estimated a recording radius of 97 μm. Thus, neuron pairs that are isolated concurrently on a single tetrode are likely to be at most ~200 μm apart. Neuron comparisons made between isolated neurons in ATs are at most ~370 μm apart (170 μm average tetrode separation and 2 × 97 μm based on the recording radius).

**Figure 2:**
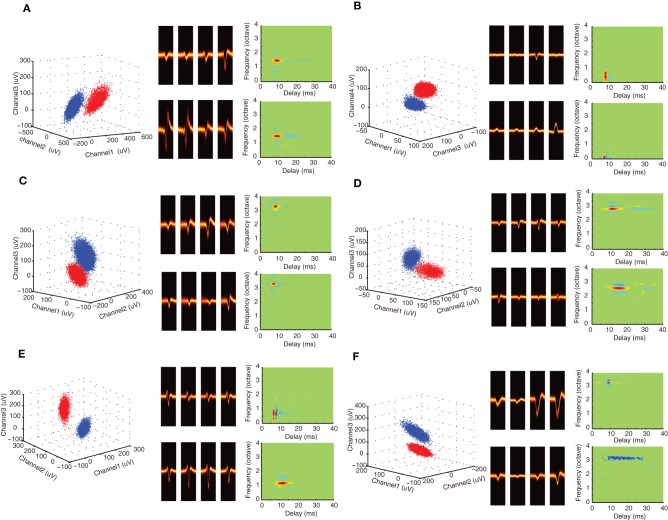
**Spectrotemporal selectivity of neighboring single neurons. (A–F)** Example single unit waveforms and the corresponding STRFs are shown for neighboring neurons. All of the examples follow the same format as Figures 1C,D. For each unit, the waveform peak amplitude clusters are shown (*left*, selected tetrode channels) along with the spike waveform density plots for the isolated units (*middle*). STRFs are shown on the *right* two panels for each isolated single unit example. Red colors indicate that the sound tended to be on prior to the occurrence of an action potential (at 0 ms delay) and are thus indicative of excitation. Blue domains indicate that the sound tended to be off and are thus indicative of inhibition or suppression. Green colors indicate that the sound had no effect on the generation of action potentials. As can be seen from the examples, neighboring neurons can have similar STRFs indicative of similar spectral and temporal selectivity (e.g., **A–D**). By comparison, other sites have STRFs that can differ substantially along the temporal and/or spectral dimensions (e.g., **E–F**).

At each recording location well-defined action potential clusters could be identified. As can be seen for the example recordings (Figures 1C,D; also Figures 2A–F), the waveform density plots for each isolated unit exhibits a consistent and unique pattern across each of the tetrode channels (middle panels). Furthermore, the peak amplitudes for selected tetrode channels form well-defined clusters (left, blue and red), which is indicative of well-isolated single units. Stringent criteria were applied to identify single units (see Materials and Methods) and in total we had 344 well-isolated single units from 123 recording sites, which resulted in 93 single unit pairs identified on a single tetrode (ST pairs) and 333 single unit pairs identified across ATs (AT pairs).

### Spectrotemporal response properties of neighboring neurons

DMR sound was delivered while recording neural responses from multiple single neurons. This dynamic stimulus contains spectral and temporal features such as temporal modulations, spectral peaks and notches that are common features in natural sounds and which efficiently drive ICC neurons (Escabí and Schreiner, 2002; Rodriguez et al., 2010a). The DMR is statistically unbiased and contains temporal modulations spanning 0–500 Hz and spectral modulations from 0 to 4 cycles/octave, which allows us to measure STRF of each neuron. The STRF is obtained as the average spectrotemporal envelope preceding all action potentials and it thus represents the spectrotemporal sound pattern that is most likely to evoke an action potential. Alternately, the STRF can be viewed as a spectrotemporal filter function that maps the sound to a neural response pattern. Positive regions in the STRF are thus indicative of excitation while negative regions indicate inhibition or suppression. As can be seen for example neuron pairs (Figures 1C,D; Figures 2A–F), the STRFs can be highly similar across neighboring neuron pairs (Figures 2A–D) or quite different (Figures 2E–F). For example, the neighboring neurons in Figure 1C have an overlapping excitatory region around 3.5 octave (all frequency measurements are performed relative to 1 kHz). Accordingly, these neurons have similar BFs (3.45 vs. 3.48 octave) and latency (6.5 vs. 7.2 ms). However, other spectral and temporal characteristics can be more varied across neighboring pairs such as the amount and strength of suppression/inhibition (blue STRF domains), which is quite different for these two units. Certain neighboring pairs have largely similar STRFs with similar BF, bandwidth and integration time (e.g., Figures 2A–D). However, at other recording sites, the receptive field structure between neighboring neurons can vary substantially (e.g., Figure 1D; Figures 2E–F). The neighbors shown in Figure 2E have similar BFs (0.71 vs. 1.17 octave), however, the latency (6.5 vs. 11.75 ms) and integration time (1.9 vs. 6.5 ms) of unit 2 are substantially longer. Furthermore, the general arrangement of excitation and inhibition are quite different for these neighboring units: unit 1 has an on–off temporal pattern while unit 2 exhibits strong lateral inhibition. For the pair in panel **F**, BFs are similar (3.3 vs. 3.2), however, unit 1 is fast and predominantly excitatory while unit 2 has a long-lasting and predominantly inhibitory structure (3.9 vs. 14.5 ms integration time). Thus for some recording sites the STRF of neighboring neurons can be quite similar while for other sites the receptive field structure can be more varied.

To characterize how much of the receptive field similarity between neighboring neurons is due to the proximity and BF match we grouped the data into five categories in the subsequent sections (see Materials and Methods). AT pairs are obtained from the same recording site but on different tetrodes while ST pairs consist of neurons isolated on the same tetrode. Distant site pairs (DS) serve as a reference control designed to account for the receptive field variability between ICC pairs that is expected by chance. Finally, the ST frequency matched group (STBF) and DS frequency matched control group (DSBF) were designed to control for the fact that certain receptive field parameters can depend strongly on BF (Joris and Yin, 1992; Rodriguez et al., 2010b) irrespective of proximity.

### Clustering of best frequencies between neighboring neurons

**Figure 3:**
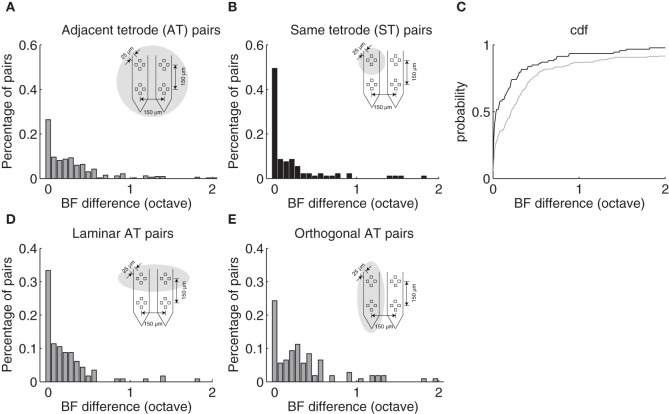
**Best frequency differences between neighboring neurons depend on proximity. (A)** BF difference distribution for neuron pairs isolated on adjacent tetrodes (AT) with a separation of 150 μm as illustrated in Figure 1B. Eighty percent of AT pairs fall within 0.55 octave. **(B)** The distribution of BF differences for neuron pairs isolated on the same tetrode (ST) are largely confined to 0.32 octave (80%). **(C)** BF difference cumulative distribution function for same (*black*) and adjacent (*gray*) tetrode neuron pairs. **(D)** BF difference distribution for neuron pairs isolated on adjacent tetrodes that are along the ICC lamina orientation. **(E)** The distribution for neuron pairs isolated on adjacent tetrodes that are orthogonal to the lamina orientation. A significant mode is observed at ~0.28 octave consistent with the average BF difference between adjacent laminae.

ST and AT neighboring neurons exhibited similar frequency selectivity and were generally closely matched in BF. The BF difference distribution is shown for AT and ST pairs (Figure 3). Although, BF difference could be as large as 3.3 and 4.1 octave for a small subset of ST and AT pairs (>2 octave for 3 and 8%; data not shown), respectively, most pairs were closely matched in BF. For both groups, BF differences were tightly clustered around 0 octave difference as can be seen by the mode near the origin in the distributions (Figures 3A,B). A large incidence of pairs had a BF difference <0.1 octave although the proportion of pairs where this was true was higher for the ST group (ST: 58%; AT = 36%). For ST pairs 80% fell within 0.32 octave whereas 80% of the AT pairs fell within 0.55 octaves of each other (Figure 3C). Furthermore, the average frequency difference for ST and AT groups was 0.28 and 0.48 octave, respectively. Thus, ST pairs are more closely matched in BF than AT pairs.

Evidence for the laminar organization was evident when we conditioned AT pairs according to the recording geometry. Given the four-pronged geometry of the tetrode array (Figure 1B) and the orientation of the electrode penetrations (~30 relative to sagittal; ~ orthogonal to the frequency band lamina), it is expected that AT pairs found on tetrodes oriented orthogonal to the frequency band lamina would differ more in BF than pairs recorded from tetrodes oriented along a lamina. As can be seen from the distribution of BF differences, orthogonal AT pairs (Figure 3E) exhibit a mode around 0.28 octave BF difference which is not present for the laminar AT pairs (Figure 3B). BF differences were bimodally distributed for orthogonal AT pairs (Figure 3E; Hartigan Dip test, *p* < 0.05) but not for laminar AT pairs (Figure 3D; Hartigan Dip test, *p* = 0.33, NS). This disparity is consistent with the idea that orthogonal AT pairs are more likely to sample neurons from neighboring frequency band lamina which have a reported frequency difference of ~0.28 octave in the cat (Schreiner and Langner, 1997) and a spatial separation comparable to the AT separation (150 μm) (Rockel and Jones, 1973; Oliver and Morest, 1984).

### Spectral response preferences are more similar for neighboring neurons than for those at distant sites

**Figure 4:**
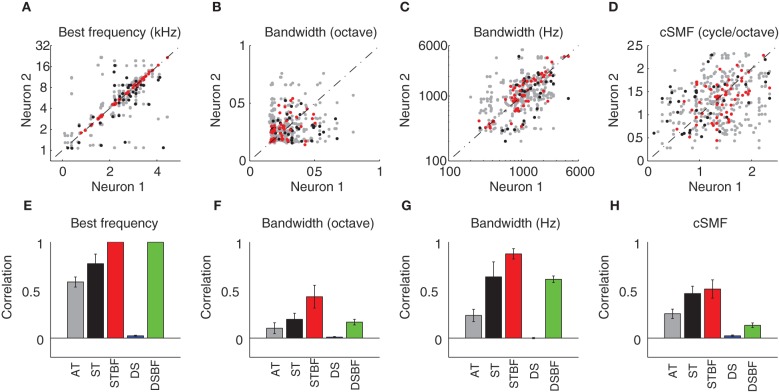
**Neighboring ICC neurons share similar spectral preferences. (A–D)** Scatter plots showing the spectral STRF parameters (**A**, Best Frequency; **B**, Bandwidth in octave; **C**, Bandwidth in Hz; **D**, cSMF) of neighboring single units (neuron 1 and 2). For each of the STRF parameters the data was partitioned into three experimental groups that depend on the spatial proximity and BF match. The experimental groups consist of neuron pairs found on adjacent tetrodes (AT, *gray*; 150 μm tetrode separation), neuron pairs detected on the same tetrode (ST, *black*), and same tetrode pairs that were matched in best frequency (STBF, *red*; <0.1 octave BF difference). **(E–H)** The Pearson correlation coefficient was used to compare the similarity for each of the spectral parameters between neighboring neurons. The measured correlation is shown for each of the experimental groups (AT, ST, and STBF) as well as for two reference controls. The reference controls consist of pairs recorded from distant sites (DS, *blue*) or distant site pairs that where matched in frequency (DSBF; <0.1 octave BF difference). Statistics for the spectral parameter comparisons are provided in Table 1. The error bars in **E–H** represent the SE of the correlation coefficient.

Spectral and temporal response properties have been shown to vary systematically within and across the frequency-band laminae in the ICC (Schreiner and Langner, 1988; Langner et al., 2002; Ehret et al., 2003; Rodriguez et al., 2010b; Baumann et al., 2011). This and anatomical data predict that response similarity would drop-off with distance between neuron pairs. We tested this prediction by measuring the similarity between spectral response properties for neighboring vs. distant pairs of neurons in each of our experimental (Figure 4; AT = gray; ST = black, and STBF = red) and control (Figure 4; DS = blue; DSBF = green) groups described above. Scatter plots for spectral STRF parameters of neighboring neurons are shown in Figures 4A–D and the resulting statistics for each of the experimental and control groups are shown in Figures 4E–H. As expected in the ICC, neuron pairs recorded on the ST had highly correlated BFs while those on ATs (more distant on average) were slightly less so (DS<AT<ST, *p* < 0.001; Figures 4A,E). Not surprisingly, randomly selected neuron pairs from different recording sites (DS group) were not correlated on average.

Spectral bandwidth varies systematically within and across the frequency-band laminae (Schreiner and Langner, 1988; Rodriguez et al., 2010b) leading us to test the prediction that bandwidth similarity would drop-off with distance. Spectral bandwidths (in octaves; Figures 4B,F) were more correlated for the AT group compared to DSs (Figure 4F; Table 1). This correlation systematically increased with proximity (DS vs. AT, *p* < 0.01) and BF match (ST vs. STBF, *p* = 0.017) and the resulting correlation is stronger than the BF matched control group (STBF vs. DSBF, *p* = 0.002, Table 1). This suggests that, on average, relative bandwidths are more similar between neighboring neurons than for DSs even when BF match is taken into account. We also measured the similarity for absolute bandwidths (in Hz; Figures 4C,G; Table 1) since relative and absolute bandwidths have distinct trends and organizations within the ICC: absolute bandwidths tend to increase while relative bandwidths tend to decrease with increasing BF (Rodriguez et al., 2010b). As for relative bandwidths, absolute bandwidths exhibit similar trends, however, the correlation for the DSBF control group was substantially higher likely reflecting the fact that absolute bandwidth are strongly correlated with BF. Despite this residual correlation, the ST frequency matched group (STBF) were more correlated on average (STBF vs. DSBF; Figure 4G, *p* < 0.001, Table 1) indicating that some of the correlation is strictly do to the proximity of neurons. Furthermore, absolute bandwidth correlation increased with decreasing distance (DS<AT<ST, *p* < 0.001).

**Table 1:** **Spectral receptive field similarity between neighboring neurons and their dependence on proximity and frequency match**.

	**DS vs. AT (*df* = 22,509)**	**AT vs. ST (*df* = 424)**	**ST vs. STBF (*df* = 145)**	**DSBF vs. STBF (*df* = 2015**)
BW (octave)	*Z* = 1.7	*Z* = 0.80	*Z* = 1.5	*Z* = 2.1
	*p* = 0.009	*p* = 0.13/N.S.	*p* = 0.017	*p* = 0.002
BW (Hz)	*Z* = 4.3	*Z* = 4.3	*Z* = 3.5	*Z* = 4.6
	*p* < 0.001	*p* < 0.001	*p* < 0.001	*p* < 0.001
cSMF	*Z* = 4.2	*Z* = 2.0	*Z* = 0.35	*Z* = 3.0
	*p* < 0.001	*p* = 0.002	*p* = 0.31/N.S.	*p* < 0.001

Statistics are shown for the receptive field bandwidth (in octave and Hz) and the characteristic spectral modulation frequency (cSMF). The correlation coefficient between the parameters of neurons pairs was estimated for each of the experimental and control groups (data shown in Figure 4). Statistics for the comparisons between experimental (AT, ST, and STBF) and control groups (DS and DSBF). Comparisons of the correlation coefficient between groups were made at a chance level of 0.05 and were corrected for the number of comparisons (Bonferroni correction). Non-significant comparisons are noted by N.S.

We also tested whether selectivity for spectral modulations was similar for neighboring neurons. Spectral modulations are common features in natural sounds, consisting of peaks and valleys in the sound spectrum, such as for head related spatial cues (Kulkarni and Colburn, 1998) or formants in speech (Klein et al., 1970; van-Veen and Houtgast, 1985). Given that ICC neurons respond selectively to spectral modulations and their characteristic spectral modulation frequency (cSMF, see Materials and Methods) is inversely related to their BW (Qiu et al., 2003; Rodriguez et al., 2010b), we expect to see similar dependencies between BW and cSMF. As illustrated, there was an increase in the cSMF correlation with our metrics of proximity for pairs of neurons (i.e., DS<AT<ST, *p* < 0.01; Figures 4D,H; Table 1); though the trend was less pronounced than that associated with BW and BF match did not have a significant effect (Figure 4H, ST vs. STBF, *p* = 0.31). However, the correlation for STBF group is much higher than the DSBF (*p* < 0.001). This indicates that spectral modulation preferences are more similar among local neighboring neurons pairs even when BF match is accounted for.

### Temporal response preferences are more similar for neighboring neurons than for those at distant sites

**Figure 5:**
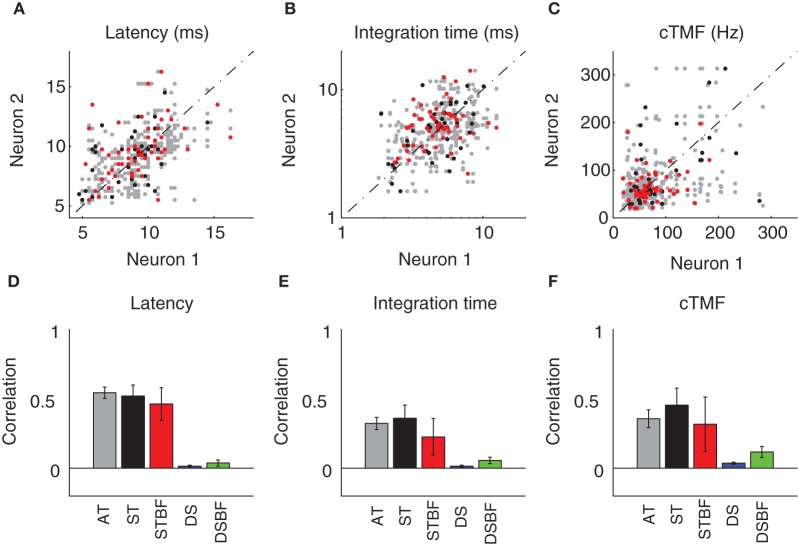
**Neighboring ICC neurons share similar temporal preferences. (A–D)** Scatter plots showing the temporal STRF parameters (**A**, Latency; **B**, Duration; **C**, cTMF) of neighboring single units (neuron 1 and 2). The data was partitioned into three experimental groups as in Figure 4 (AT, *gray*; ST, *black*; STBF, *red*). The Pearson correlation coefficient was used to compare the similarity for each of the temporal parameters between neighboring neurons. The correlation coefficient is shown for each of the experimental groups as well as for two reference controls (DS, *blue*; DSBF, *green*). Statistics for the temporal parameter comparisons are provided in Table 2. The error bars in **D–F** represent the bootstrapped SE of the correlation coefficient.

Temporal response properties in the ICC vary systematically with position within and across the frequency-band laminae (Schreiner and Langner, 1988; Langner et al., 2002; Middlebrooks and Snyder, 2010; Rodriguez et al., 2010b). Once again, this response organization leads us to hypothesize a drop off in the similarity of temporal response properties between neuron pairs with distance. The first temporal response property we considered, STRF latency, was the most correlated temporal parameter between neighboring neurons (Figures 5A,D). STRF latencies were quite varied across our sample and confined mostly between 5 and 15 ms. Correlations for neighboring pairs in a recording site were significantly higher than those from distant recording sites (DS vs. AT, *p* < 0.001, Table 2) regardless of whether the pairs had matched BF or not (DSBF vs. STBF, *p* < 0.001, Table 2). However, within the radius of the tetrode array there was not a significant relationship between the latencies of neighboring neurons as a function of proximity or BF match (AT vs. ST; ST vs. STBF, Table 2, N.S.).

**Table 2:** **Temporal receptive field similarity between neighboring neurons and their dependence on proximity and frequency match**.

	**DS vs. AT (*df* = 22,509)**	**AT vs. ST (*df* = 424)**	**ST vs. STBF (*df* = 145)**	**DSBF vs. STBF (*df* = 2015)**
Latency	*Z* = 10.7	*Z* = −0.27	*Z* = −0.44	*Z* = 3.3
	*p* < 0.001	*p* = 0.35/N.S.	*p* = 0.27/N.S.	*p* < 0.001
IT	*Z* = 7.1	*Z* = 0.53	*Z* = −0.92	*Z* = 1.8
	*p* < 0.001	*p* = 0.23/N.S.	*p* = 0.10/N.S.	*p* = 0.005
cTMF	*Z* = 6.1	*Z* = 0.98	*Z* = −0.92	*Z* = 1.5
	*p* < 0.001	*p* = 0.08/N.S.	*p* = 0.09/N.S.	*p* = 0.018/N.S.

Statistics are shown for the receptive field latency (ms), integration time (ms), and the characteristic temporal modulation frequency (cTMF). Tests for significant differences of the parameter correlation coefficients (Figure 5) were performed and are reported between the experimental (AT, ST, and STBF) and control groups (DS and DSBF). All comparisons were made at a chance level of 0.05 and were corrected for the number of comparisons (Bonferroni correction). Non-significant comparisons are noted by N.S.

A similar trend was observed for the STRF integration time (IT; Figures 5B,E; Table 2). The IT represents the time window over which the sound history has a significant effect on the neuron's response (Chen et al., 2012). For most neurons, ITs were confined between 2 and 10 ms implying that temporal features in the DMR of at most 10 ms duration had a direct effect on the neuron's response. Within a local neighborhood, ITs were more similar than for DSs (AT, ST vs. DS, *p* < 0.001; Figures 5B,E, Table 2), even when BF match is taken into account (STBF vs. DSBF, *p* = 0.005). However, a significant trend was not observed with increasing proximity and BF match (AT vs. ST; ST vs. STBF, Table 2, N.S.), indicating that the IT similarity amongst neighboring neuron does not vary substantially within a local neighborhood.

Temporal modulation preferences are also organized systematically within and across the frequency-band laminae (Schreiner and Langner, 1988; Rodriguez et al., 2010b). Here, the relationship between temporal modulation preferences and proximity was determined by computing the characteristic temporal modulation frequency (cTMF) of each neuron (see Materials and Methods). As for the other temporal STRF parameters, cTMFs were more similar between neighboring neurons compared to DSs (AT vs. DS, *p* < 0.001; Figures 5C,F, Table 2). cTMFs have also been shown to vary with BF (Rodriguez et al., 2010b), however, even when BF dependence is taken into account neighboring neurons were still more similar than distant and BF matched sites (STBF vs. DSBF, *p* = 0.018; Figures 5C,F, Table 2). However, unlike spectral modulation sensitivity where neighboring neurons were more similar as a function of local proximity and BF match in a local neighborhood, we did not observe such effects for cTMFs (AT vs. ST; ST vs. STBF, N.S.; Figures 5C,F, Table 2).

### Receptive field and spike train correlation of neighboring neurons

The results from the previous analysis demonstrated that spectral and temporal receptive field characteristics are relatively more similar within a local neighborhood. One may thus be led to believe that the transmitted signals to the auditory thalamus from neighboring neurons would be highly correlated, which could indicate a form of redundant information transfer. However, the analysis employed in the previous section strictly examines the preferred sensory features of neighboring neurons and tells us little about the temporal response pattern for each particular feature. For instance, two neurons may respond to similar sound features on average (as noted by their STRFs). However, the same average STRF can be obtained by summing different (even non-overlapping) subsets of elements so that the individual features that evoke spikes for each neuron can be quite different from one action potential to the other (Escabi et al., 2005). Thus, the temporal patterning of the resulting spike trains to a sound for each neuron could be vastly different even when neurons have similar STRFs (Chen et al., 2012). Thus, it is theoretically possible that, although ICC neurons share similar preferences in a local neighborhood, their temporal responses are uncorrelated so that each neuron conveys independent sensory information to the thalamus.

**Figure 6:**
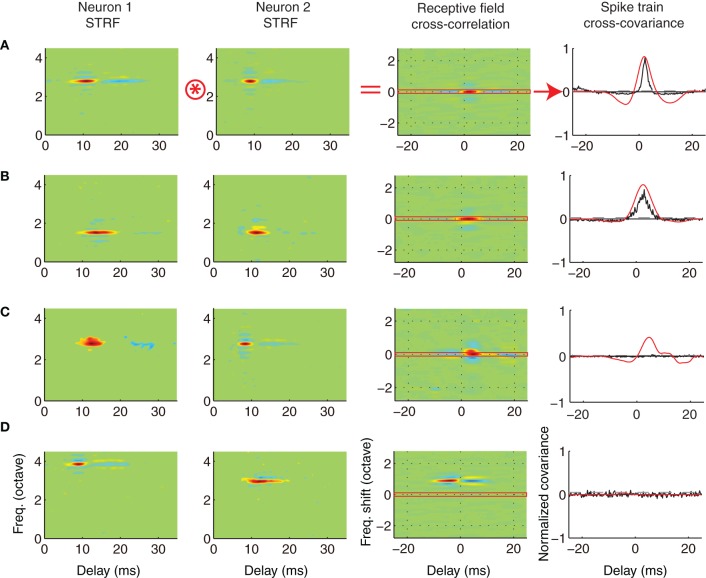
**Relationship between the receptive field and spike train crosscovariance of neighboring and distant neurons.** The STRFs of four example neuron pairs are shown (**A–D***, columns 1 and 2*). The examples were chosen from the experimental (**A**, ST; **B**, AT) and control groups (**C**, DS; **D**, DSBF). For each pair, we computed the spike train crosscovariance function (SCC, normalized between -1 and 1, see Materials and Methods) to determine the strength and timing of the temporal correlations between the spike trains of neighboring neurons (*column* 4, *black*). We used the STRFs for each pair to predict the SCC. For each pair of neurons, this is done by performing a two-dimensional cross-correlation between the STRF of each neuron (*column* 3) and subsequently selecting the temporal cross-section about zero frequency shift (red horizontal section, in *column* 3; see Materials and Methods). For instance, the example neuron pairs of **A** and **B** have STRFs with similar structure and thus the RFCC shows a strong positive peak about zero frequency shift. The predicted spike train covariance mirrors the SCC for each pair (column 4, *red*). In **C**, the neurons are highly overlapped in frequency, however, STRFs fail to predict the lack of correlation observed between the neural spike trains. The pair shown in **D** has receptive fields that are temporally delayed (neuron 1 leads neuron 2) and which differ in frequency by 1 octave. Because of the lack of frequency overlap the predicted (*red*) and measured SCC (*black*) are both zero.

To examine how sound information is represented by ICC neuron pairs we examined how the receptive field of neighboring neurons contributes to correlated activity. As a measure of receptive field similarity we estimated the RFCC (RFCC; see Materials and Methods) between pairs of neighboring (Figure 6; **A** = ST; **B** = AT) and distant neurons (**C** = DSBF; **D** = DS). Example STRFs are show for four pairs of neurons (Figure 6) along with the resulting RFCC for each pair. The RFCC represents the stimulus driven correlations that are shaped by the spectral and temporal integration properties of each neuron. Neighboring neurons pairs with similar STRFs have a strong localized peak in the RFCC about zero frequency shift (Figures 6A,B; 3rd panel), indicating a high degree of spectral and temporal receptive field alignment. In contrast, the RFCC for a distant pair with frequency mismatched STRFs have a displaced peak at ~1 octave frequency separation (Figure 6D, 3rd panel), indicative of their frequency mismatch.

For each of the neuron pairs we next measured the amount of correlated activity in their neural spike trains by computing the normalized shuffled spike train cross covariance (SCC) (Figure 6; right panel, black lines). The RFCC was then used to predict the SCC. This is done by selecting the RFCC cross-section about zero frequency shift (Figure 6, right panel, red lines). As illustrated, the SCC is smaller in magnitude than the prediction based on the RFCC (Figures 6A,B, right panel) or it can be completely absent, as for the BF matched distant pairs (Figure 6C). Thus the RFCC sets an upper bound on the SCC (Chen et al., 2012). Furthermore, when there are strong spike train correlations as for the neighboring pairs of Figures 6A,B, the RFCC faithfully predicts the timing of the SCC. If neighboring neurons had a linear stimulus-response relationship the SCC and the prediction based on the RFCC would be identical. The observed reduction in the correlation strength between the neuron's integration process (RFCC) and the output spike train (SCC) could theoretically reflect factors that are specific to each neuron, such as intrinsic response variability (“neural noise”) or output non-linearities in the spike generating mechanisms (Chen et al., 2012).

**Figure 7:**
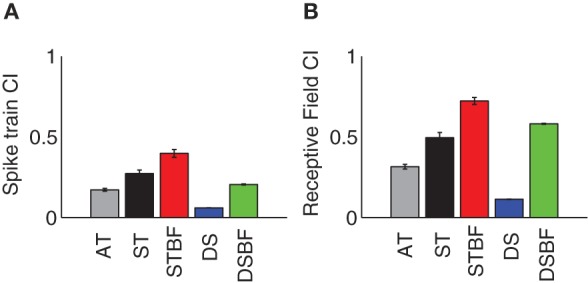
**Receptive field and spike train correlation statistics for neighboring ICC neurons.** The average absolute spike train CI **(A)** and receptive field CI **(B)** both increases with proximity (AT, *gray* vs. ST, *black*) and BF match (ST, *black* vs. STBF*, red*). All examples are shown for three experimental groups (AT, *gray*; ST, *black*; STBF, *red*) and two control groups (DS, *blue*; DSBF, *green*). The error bars represent the bootstrapped SE.

We asked whether receptive field similarity between neuron pairs determine the amount of correlated neural activity in their spike trains and whether these factors depend on proximity and frequency match. Given that the receptive field parameters of neighboring neurons tend to be similar within a local neighborhood, we expect that the RFCC and SCC should follow similar trends. For each pair we computed the receptive field and spike train correlation index (CI, peak of the RFCC and SCC, respectively) and the results were analyzed as a function of proximity and frequency match (Figure 7, see Table 3 for statistics). As can be seen, both the receptive field and spike train CI are larger for neighboring neurons compared to the distant control group (AT vs. DS, Figures 7A,B; Table 3). Both the receptive field and spike train CI increase with proximity (AT vs. ST, *p* < 0.001; Figures 7A,B; Table 3) and frequency match (ST vs. STBF, *p* < 0.001; Figures 7A,B; Table 3). Furthermore, these effects were not simply the result of frequency match between neighboring neurons since the receptive field and spike train CI of distant neurons with matched frequency was significantly lower than for frequency matched neighboring neurons (STBF vs. DSBF, *p* < 0.001; Figures 7A,B; Table 3).

**Table 3:** **Response and receptive field correlation between neighboring neurons and their dependence on proximity and frequency match**.

	**DS vs. AT (*df* = 22,509)**	**AT vs. ST (*df* = 424)**	**ST vs. STBF (*df* = 145)**	**DSBF vs. STBF (*df* = 2015)**
RFCI	*T* = 20.5	*T* = 6.1	*T* = 4.8	*T* = 6.6
	*p* < 0.001	*p* < 0.001	*p* = <0.001	*p* = <0.001
STCI	*T* = 23.1	*T* = 4.8	*T* = 3.5	*T* = 7.8
	*p* < 0.001	*p* < 0.001	*p* = <0.001	*p* = <0.001

Statistical comparisons are reported for the receptive field correlation (RFCI) and spike train correlation index (STCI). Test for significant differences were performed between the experimental (AT, ST, and STBF) and control groups (DS and DSBF; data shown in Figure 7). All comparisons were made at a chance level of 0.05 and were corrected for the number of comparisons (Bonferroni correction). Non-significant comparisons are noted by N.S.

## Discussion

The synaptic domain hypothesis predicts the brainstem inputs to the ICC cluster into zones with common anatomic input and similar sounds preference (Oliver, 2000; Loftus et al., 2010). Here, we have demonstrated that neurons are highly localized into functional zones with similar spectrotemporal sensitivity, supporting the domain hypothesis organization within the ICC.

### Relationship between micro and global organization

Spectral and temporal preferences of neighboring neurons were highly organized within the recording radius of the tetrode array; yet these organizations were subtly different for spectral and temporal preferences. For both spectral and temporal receptive field parameters, correlations between neighboring neurons were substantially larger than for DSs. This was true even when distant recording pairs were compared with matched BF suggesting that the observed correlations are not simply due to frequency dependence of response properties. Spectral properties were the most similar between neighboring neurons and the spectral receptive field similarity increased with proximity and BF match. This suggests that spectral properties are highly localized into zones within a recording radius of at most ~100 μm. In contrast, no such effects were observed for the temporal receptive field parameters (Figure 5). Temporal preferences were similarly correlated for ST (100 μm radius) and AT (185 μm radius) comparisons. This implies that temporal preferences were relatively homogeneous over a larger recording radius implying that the size of the “neighborhood” is more extensive for temporal than for spectral response preferences.

Clustering for BF was observed for AT pairs oriented orthogonal to the anatomical laminar organization (Figures 3D,E) consistent with the hypothesis that frequency organization is discretized within the ICC (Schreiner and Langner, 1997; Malmierca et al., 2008). However, no such clustering was observed for other spectral and temporal response properties (data not shown). All of the correlations for spectral and temporal parameters were not significantly different for orthogonal vs. laminar AT pairs (Fisher z-transform test, all comparisons NS). This supports the hypothesis that adjacent laminae perform similar functions and thus process similar acoustic cues.

Several organizational schemes could lead to the observed response patterns observed here. First, spectral and temporal response properties are topographically ordered within and across the ICC laminae. Temporal properties have been shown to have a gradient organization along the medial and lateral aspect of the IC (Schreiner and Langner, 1988; Langner et al., 2002) while spectral preferences seem to have a concentric organization within a lamina (Schreiner and Langner, 1988; Ehret et al., 2003). Spectral and temporal sensitivities also vary systematically along the dorso-ventral extent and are thus highly correlated with the frequency organization (Rodriguez et al., 2010b). Regardless of whether the organization is patchy or continuous, our results support a scheme where local networks of neurons share a common organizing principle as inferred from the local similarities in the sound preferences.

### BF difference and laminar organization

The measured BF differences from neighboring neurons are quantitatively consistent with previously reported frequency-band laminar organization, which is the primary organizing principle within the central nucleus. Adjacent laminae are reported to be ~150 μm apart in the cat ICC (Rockel and Jones, 1973; Oliver and Morest, 1984) and have reported frequency separation of ~0.28 octave (Schreiner and Langner, 1997).

Based on our estimated recording radius for ST pairs of 100 and 185 μm for AT pairs, and the measured BF differences we estimate that most of our recordings fall within the bounds of two or three adjacent laminae for ST and AT groups, respectively. The ratio of the estimated recording radius for ST vs. AT pairs is 100/185 = 0.54 is similar to the ratio of the average BF difference for the same groups, 0.28/0.48 = 0.58. Thus the spread of the measured BF distribution increases proportional to the recording radius. For ST comparisons, the estimated recording radius of 100 μm implies that neurons were at most 200 μm apart, which implies that neighboring neurons were either within the same lamina or one lamina apart. The average BF difference of 0.28 octave for ST comparisons fits with this expectation since it matches the average frequency separation between adjacent laminae in the cat (Schreiner and Langner, 1997). Furthermore, 80% of ST pairs fall within 0.32 octave which likewise supports the idea that most neurons are at most one lamina apart while 80% of AT pairs fall within 0.55 octave, which is consistent with the idea that pairs from this group could be up to two laminae apart. Finally, additional support for laminar organization is provided by the fact that BF differences for AT pairs oriented orthogonal to the laminar orientation exhibited a mode at 0.28 octave, comparable to the laminar separation, while AT pairs oriented along a lamina did not.

Although, the vast majority of neighboring neuron pairs were closely aligned in BF, consistent with the laminar separation, a small number of neuron pairs (6% of ST and 13% of AT pairs) were substantially more dispersed (i.e., more than 1 octave). Several factors could account for such large BF disparities. First, the estimated recording radius represents the average radius based on the statistical measurements from the recordings. For any given neuron, the recording radius actually depends on the strength of the source current, which scales proportional to the neuron size (Mechler et al., 2011). Within the ICC, disk shaped cells are the most prominent and have relatively small cell bodies with flattened dendrites that are confined to a single anatomical lamina while stellate cells are less prevalent but they have a substantially larger soma. Thus, it may be expected that disk shaped cells, which are the most abundant, will have a relatively small recording radius and BF differences between these neuron types will be relatively small as for most of our recordings. The recording radius of the more sparsely distributed stellate cells, however, should be substantially larger, which could explain the large BF disparities observed for a small number pairs. This possibility is supported by the fact that pairs with large BF differences (>1 octave) had highly similar temporal properties (delay: *r* = 0.75 ± 0.05, *p* < 0.001; integration time: *r* = 0.47 ± 0.07, *p* < 0.001; cTMF: *r* = 0.55 ± 0.1, *p* < 0.001) but differed vastly in spectral properties (bandwidth: *r* = −0.16 ± 0.13, N.S.; cSMF, *r* = 0.25 ± 0.1, N.S.). Secondly, across-lamina integration (Biebel and Langner, 2002) could also potentially account for the large disparities observed. Stellate cells can have extensive collaterals that can cross and integrate across multiple frequency laminae (Oliver et al., 1991). Inputs from distant collaterals could thus potentially shift the BF of a neuron relative to that of its local neighbors.

### Receptive field correlation and correlated firing: implications for downstream signaling

The structure of the sensory information relayed to the thalamus ultimately depends on the spike train output from ICC onto recipient thalamic neurons. The amount, strength, and type of correlated activity between converging ICC neurons can potentially enhance the fidelity of the transmitted message or alternately limit the amount of transmitted information due to a high degree of redundant firing.

Several functional rules were identified for the structure of correlated activity between neighboring ICC neurons. First, the receptive field and spike train correlation between neighboring neurons increases with proximity and BF match (Figures 7A,B) so that neighboring neurons have more similar receptive fields and more strongly correlated firing patterns compared to DSs. This supports the idea that neighboring neurons are organized into functional zones that preferentially respond to certain spectrotemporal features.

An intriguing aspect of these finding is the dependence of correlated firing on ~1/3 octave spectral separation. Significant correlated firing is not found outside this range (Chen et al., 2012) and here we have shown a second dependence on the spatial separation of neighboring neurons. This contrasts with a previous study where correlated activity was pervasive within the IC for sequentially recorded neurons that could differ vastly in their BF (Chechik et al., 2006). One factor that potentially contributes to this disparity is the fact that the natural sounds used in that study where synthetically shifted in frequency for some neurons to match their BF. Under such conditions neurons with different BFs would be driven with the same sound envelope even if the original natural sound envelopes for each neuron are uncorrelated. This could artificially increase the likelihood of observing correlated firing between neurons. Furthermore, the natural sounds used consisted of a limited subset of slow but transient bird vocalizations with highly restricted spectrotemporal variation. The sharp transients in those sounds presented against silence could potentially drive onset activity in the ICC (Zheng and Escabi, 2008), which could amplify the amount of correlation between neurons. This contrasts with the DMR, which extensively probes the modulation space and, in particular, extends to high modulation frequencies (up to 500 Hz) that IC neurons can selectively respond to. The biased sound repertoire in that study could likewise exacerbate the amount and strength of correlated activity.

Non-linearities in the spike generating mechanisms can impact the amount and strength of correlated activity between neurons. As seen for the data, the receptive field CI on average exceeded the spike train CI, implying that non-linearities may contribute to the low levels of spike train correlation between neurons. Intriguingly, for some neuron pairs spike train correlations are not present even though the receptive field correlations was strong (e.g., Figure 6C). Thus, two neurons can respond to similar sound patterns on average even if their spike times are temporally uncorrelated. This may seem surprising, but is consistent with our recent findings demonstrating that non-linearities in the spike generating mechanism can decorrelate neural spike trains between neurons that have similar BF and receptive field preferences (Chen et al., 2012). Thus, differences in the dynamics of the spike generation, intrinsic noise, and adaptive non-linearities can all potentially reduce the amount and strength of spike train correlation. As observed, the spike train CI of nearby neuron pairs with matched BF (STBF) was substantially larger than that of DSs with matched BF (DSBF). Thus, it is possible that neighboring neurons share similar types of non-linearities while more distant neurons have uniquely different non-linear response properties, which would ultimately reduce the strength of correlated activity between distant neurons.

Anesthesia and binaural interactions can also potentially impact the correlation strength. Anesthesia would have a tendency to reduce driven firing rates, which would in turn produce lower correlation values (de la Rocha et al., 2007). However, this is unlikely to affect the observed trends between neighboring and distant neurons. As demonstrated, the RFCC and SCC exhibited similar trends as a function of the proximity and BF match between neurons although the magnitude of the SCC was smaller (Figures 7A,B). This is consistent with the fact that RFCC serves as a upper bound for the SCC. Furthermore, we have previously demonstrated that the RFCC can predict various attributes of the SCC including the waveform shape and peak timing (Chen et al., 2012). This indicates that the resulting population trends largely reflect phase-locked stimulus driven correlations, as intended. Binaural interactions can also potentially affect the correlation between neurons since ipsilateral inputs to the ICC can have strong inhibitory influence (Oliver, 2000). Out of five experiments where we delivered sounds dichotically, only 17% of neurons exhibited significant ipsilateral STRFs and these where always weaker than the contralateral STRF. Thus, phase-locked activity originating in the ipsilateral ear is weak and likely contributed little to the SCC. This is consistent with the fact that RFCC derived from the contralateral ear STRF and SCC, which was obtained with sounds delivered dichotically, had similar trends. Furthermore, the general trends for the five experiments where we delivered sounds dichotically were the same for the remaining two experiments where we delivered sounds monaurally (data not shown). This implies that binaural interactions had little effect on the results.

There are several implications of the observed patterns of correlated activity within local IC populations. First, strongly correlated activity is potentially detrimental from a coding efficiency perspective because the neural messages relayed would be highly redundant (Barlow, 2001). However, correlated firing can be beneficial as it may allow for postsynaptic signal transmission via coincidence detection of weak synaptic inputs (Bruno and Sakmann, 2006). For instance, correlated firing between neighboring neurons in the retina has been shown to enhance information transmission in retinal ganglion cell populations (Pillow et al., 2008). Within the IC a fine balance in the amount of correlated firings seems to be achieved since most of the neural activity is uncorrelated except for a small subset of neurons pairs falling within ~1/3 octave. Strikingly this spectral separation of ~1/3 octave for correlated firing roughly corresponds to the anatomical laminar distance in cat (Schreiner and Langner, 1997) and mirrors the “critical band” which is the hallmark for perceptual resolution in humans and mammals (Fletcher, 1940; Yost and Shofner, 2009).

### Comparison to previous studies

A single previous study directly examined the response preferences between neighboring neurons in the IC of cats (Seshagiri and Delgutte, 2007). They found that neighboring single neurons recorded on a single tetrode had similar BF and bandwidths which is similar to our findings. However, many of the remaining response parameters measured in that study including the type of frequency response area and temporal response patterns were uncorrelated between neighboring neurons. This suggests that neighboring neurons are largely heterogenous within an ICC neighborhood, which contrasts our result.

One difference between the studies is that we observe substantially more correlation for temporal response properties based on our metrics. Seshagiri and Delgutte compared the type of PSTH (i.e., onset, sustained, chooper etc.) between adjacent neurons and found that the temporal response pattern to tone-burtsts (measured at CF) where randomly distributed. In our case, we performed detailed analysis by comparing temporal parameters of the STRFs and found significant correlations for all parameters measured (Figures 5, 7). Furthermore, we found that the spike trains to the DMR are correlated between neighboring neurons and these correlations increased with proximity indicating that the firing pattern of neighboring neurons are similar. Seshagiri and Delgutte also compared the patterns of excitation and inhibition in the frequency response maps of neighboring neurons using the classifications of Ramachandran et al. (1999) and found these to be randomly distributed throught the IC. Although, we did not categorize neurns into specific types based on excitation and inhibition, we note that cSMF and cTMF measurements depend strongly on spectral and temporal inhibitory patterns (Rodriguez et al., 2010b) and both of these parameters where significantly correlated. Finally, Seshagiri and Delgutte did not observe clustering of BF differences whereas we observed clustering for AT recordings (Figure 3).

The simplest explanation for the differences between these studies is that they are due to differences in the tetrode recording configuration. Based on our (100 μm) and their reported estimates (125 μm) of the recording radius this is unlikely a factor. One possible explanation is that the spectro-temporal parameters we tested are more in line with the organizing principles of the IC. In particular, all of the metrics employed here are based on phase-locked neural responses to the stimulus envelope while most of the measures reported in the earlier study are based on average firing rates. The types of stimuli employed are distinctly different between the two studies and likely contribute to the differences. The DMR sound employed here dynamically activates the entire sensory epithelium and, because of its broadband structure, concurrently activates excitatory and inhibitory circuits within and outside the IC. By comparison, Seshagiri and Delgutte measured pure-tone response properties that only activate a restricted portion of the sensory network and do not sample sound modulation preferences that have been shown to be organized within the IC. In particular, spectral and temporal resolution and modulation parameters similar to those employed here are systematically ordered within the ICC lamina as well as across the frequency dimension (Schreiner and Langner, 1988; Rodriguez et al., 2010b; Baumann et al., 2011).

Our extensive sampling across the rostral-caudal, medio-lateral, and dorso-ventral dimensions may assure that there is sufficient variation in the response properties, which is necessary to observe correlations between neighboring neurons. In our case, electrode penetrations where oriented roughly orthogonal to the frequency lamina (~30 relative to the sagittal axis) while Seshagiri and Delgutte employed a caudal-to-rostral trajectory where penetration tracks course roughly parallel to the ICC frequency band lamina. As a result, that study focused primarily on low frequency recording locations (mostly 0.5–2 kHz) while our study focus on higher frequencies (97% of neurons within 1–16 kHz), which likely contributes to some of the differences between the studies. Given our extensive sample we defined two controls (DS and DSBF) that set a reference point for defining a baseline level of receptive field and spike train correlation. This scheme enabled us to demonstrate that some of the correlation between neighboring neurons is due to BF match (irrespective of proximity) while the remaining correlation is accounted by the recording proximity (i.e., distant vs. neighboring sites). Finally, the four-tetrode configuration used in this study enabled us to compare neurons found on the ST vs. those found on AT. Using this scheme we demonstrate that even within the small recording radius of the tetrode array (~185 μm for AT pairs; ~100 μm for ST pairs) receptive field correlations can further increase with the local proximity of neurons indicating that neuron preferences cluster into relatively small neighborhoods with highly similar response preferences.

### Conflict of interest statement

The authors declare that the research was conducted in the absence of any commercial or financial relationships that could be construed as a potential conflict of interest.
